# Sorting Nexin 9 facilitates podocin endocytosis in the injured podocyte

**DOI:** 10.1038/srep43921

**Published:** 2017-03-07

**Authors:** Yu Sasaki, Teruo Hidaka, Takashi Ueno, Miyuki Akiba-Takagi, Juan Alejandro Oliva Trejo, Takuto Seki, Yoshiko Nagai-Hosoe, Eriko Tanaka, Satoshi Horikoshi, Yasuhiko Tomino, Yusuke Suzuki, Katsuhiko Asanuma

**Affiliations:** 1 Division of Nephrology, Department of Internal Medicine, Juntendo University Faculty of Medicine, Tokyo, Japan; 2 Laboratory of Proteomics and Biomolecular Science, Research Support Center, Juntendo University Graduate School of Medicine, Tokyo, Japan; 3 Medical Innovation Center, TMK project, Graduate School of Medicine, Kyoto University, Kyoto, Japan; 4 Department of Pediatrics and Developmental Biology, Graduate School of Medicine, Tokyo Medical and Dental University, Tokyo, Japan

## Abstract

The irreversibility of glomerulosclerotic changes depends on the degree of podocyte injury. We have previously demonstrated the endocytic translocation of podocin to the subcellular area in severely injured podocytes and found that this process is the primary disease trigger. Here we identified the protein sorting nexin 9 (SNX9) as a novel facilitator of podocin endocytosis in a yeast two-hybrid analysis. SNX9 is involved in clathrin-mediated endocytosis, actin rearrangement and vesicle transport regulation. Our results revealed and confirmed that SNX9 interacts with podocin exclusively through the Bin–Amphiphysin–Rvs (BAR) domain of SNX9. Immunofluorescence staining revealed the expression of SNX9 in response to podocyte adriamycin-induced injury both *in vitro* and *in vivo*. Finally, an analysis of human glomerular disease biopsy samples demonstrated strong SNX9 expression and co-localization with podocin in samples representative of severe podocyte injury, such as IgA nephropathy with poor prognosis, membranous nephropathy and focal segmental glomerulosclerosis. In conclusion, we identified SNX9 as a facilitator of podocin endocytosis in severe podocyte injury and demonstrated the expression of SNX9 in the podocytes of both nephropathy model mice and human patients with irreversible glomerular disease.

Kidney podocytes, which are highly specialized, terminally differentiated epithelial cells located outside of the glomerular basement membrane, serve as a final barrier to urinary protein loss^1^. Continuous injury to the podocytes induces irreversible podocyte loss and glomerulosclerosis development, leading to chronic renal failure^2^. As the number of patients with end-stage renal disease is growing worldwide^3^, it is very important to prevent or attenuate podocyte injury and thus protect the kidneys from chronic renal failure; in this context, elucidating the pathogenesis of podocyte injury is urgently needed.

Several proteins play key roles in the maintenance of podocyte integrity. In particular, slit-diaphragm proteins, such as nephrin, podocin and CD2AP, were found to be critical in the prevention of proteinuria because the mutation and inactivation of these proteins led to severe proteinuria and consequent chronic renal failure^4^. Podocin is expressed at the slit diaphragm, where it is thought to act as an intracellular scaffold protein by assembling slit-diaphragm components in lipid raft-associated microdomains^4^,^5^. Indeed, Boute *et al*. were the first to demonstrate a causative link between mutations in *NPHS2*, which encodes podocin, and childhood nephrotic syndrome^6^. In addition, NPHS2 was found to be the most frequently affected gene associated with steroid-resistant nephrotic syndrome^7^, and dysregulated podocin intracellular trafficking was observed in several diseases associated with *NPHS2* mutations^8^.

Endocytosis serves as a portal; specifically, this process involves the formation of vesicles for the internalization and retrieval of plasma membrane components or transmembrane receptors. Endocytic trafficking can be divided into two main pathways; the classic clathrin-mediated endocytic pathway and the non-classic clathrin-independent pathway^9^. To date, a great deal of work has focused on clathrin-mediated endocytosis (CME), leading to an increasing understanding of the mechanisms by which the proteins involved in this process recruit cargo into developing clathrin-coated pits and subsequently form clathrin-coated vesicles^10^. Although CME is certainly an extremely important endocytic mechanism that accounts for a large proportion of endocytic events, an ever-expanding array of cargo has been shown to undergo endocytosis in a clathrin-independent manner^11^.

Recent findings from studies on genetic mouse models of disease, as well as human genetic mutations that result in the loss of integrity of the glomerular filtration barrier, suggest a critical role for endocytosis in podocyte biology^12^. This is because in podocytes, the endocytic process plays a fundamental role in the development and maintenance of the glomerular filtration barrier. Thus, the loss of key molecular machinery results in defective actin regulation, faulty slit-diaphragm maintenance and increases the uptake of lipoproteins, or integrins, which might have deleterious effects on podocyte health^13^.

Previously, our group has discovered a difference in the immunostaining patterns of podocin and synaptopodin, normally co-located in the foot process, in both a rat model of puromycin aminonucleoside nephrosis (PAN) and patients with IgA nephropathy^14^. In this previous study, localization of podocin shifted from the slit diaphragm to the podocyte cytoplasm in the context of severe podocyte injury, suggesting a role for endocytosis in glomerular health.

Here we describe a further novel finding that the protein sorting nexin 9 (SNX9) interacts with podocin in podocytes. SNX9 is specifically involved in endocytosis and also participates in actin rearrangement and regulates various stages of vesicle transport^15^,^16^. We demonstrate that SNX9 is strongly expressed and co-localizes with podocin in both kidneys from adriamycin (ADR)-injected mice and kidney biopsy samples from human patients with glomerular diseases leading to glomerulosclerosis. Our evidence indicates that SNX9 plays a role in podocin endocytosis and might help to advance our understanding of podocyte injury.

## Results

### The Bin–Amphiphysin–Rvs (BAR) domain of SNX9 interacts with podocin

**Figure 1:**
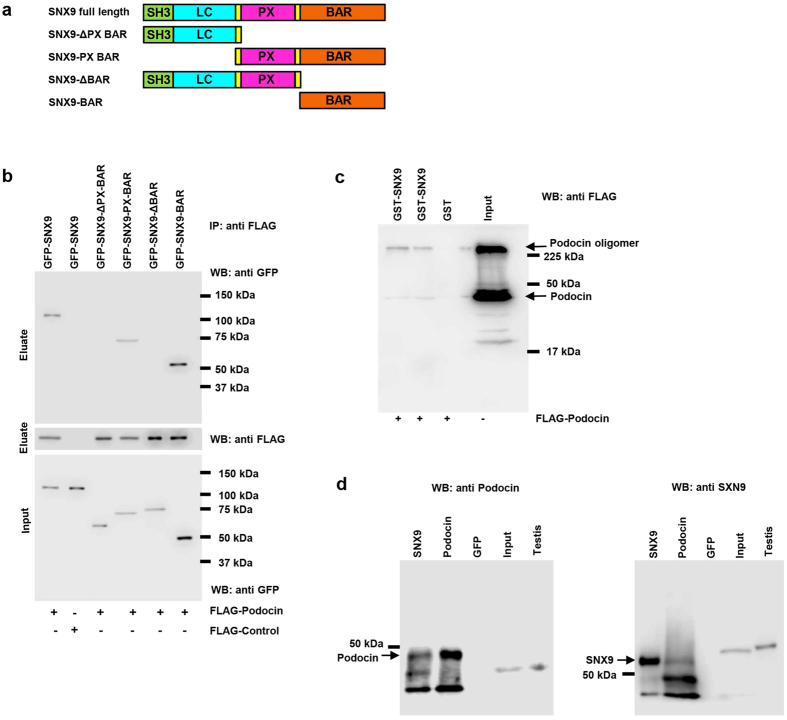
The BAR domain of SNX9 interacts with podocin. (**a**) Schematic diagram of SNX9 and its domain structure. (**b**) HEK293T cells were co-transfected with FLAG-tagged podocin and GFP-SNX9 truncated mutants; the resulting immunoprecipitates were immunoblotted with anti-GFP or anti-FLAG antibodies. The lower panel shows immunoblotting of total cell lysates with anti-GFP antibody to verify the expression of each truncated mutant. IP, immunoprecipitation. (**c**) GST–SNX9 fusion proteins, or GST alone, were incubated, and each lysate was used for the pulldown assay. Complexes were resolved by SDS-PAGE and immunoblotted with anti-FLAG antibodies. (**d**) Coimmunoprecipitation (Co-IP) experiments showing that endogenous SNX9 interacts with podocin in ADR-treated cultured human podocytes. Anti-SNX9 antibody precipitated SNX9 and coprecipitated podocin. Conversely, anti-podocin antibody precipitated podocin and coprecipitated SNX9. Anti-GFP antibody did not precipitate SNX9 and podocin.

To identify a protein that would trigger podocin endocytosis, we conducted a yeast two-hybrid screening using the C-terminal fragment of podocin as bait. We identified SNX9, a member of the sorting nexin family of proteins that is widely expressed and plays a role in endocytosis, as a novel podocin-interacting protein. Next, we conducted co-immunoprecipitation assays to confirm the interaction between podocin and SNX9 using lysates of FLAG-podocin- and GFP-SNX9-overexpressing cells and confirmed an interaction between podocin and SNX9 *in vitro*. We used HEK293T cells as podocytes which show relatively low level of transfection efficiency, typical ranging from 10% to 20% for cells grown under permissive conditions^17^, making it difficult to overexpress gene. To map the podocin binding site(s) in SNX9, we tested the abilities of various truncated GFP-SNX9 constructs^18^ to co-precipitate with FLAG-podocin (Fig. 1a,b); results show that both GFP SNX9-BAR and SNX9-PX-BAR co-precipitate with FLAG-podocin, while SNX9-ΔBAR and SNX9-ΔPX-BAR do not interact with podocin, and no SNX9 binding was observed with a FLAG control (Fig. 1b). To further demonstrate that podocin can directly bind to SNX9, we performed GST pull-down assays with purified recombinant proteins according to our previously published protocols^19^. Purified GST-SNX9, not GST alone, binds to purified FLAG-podocin (Fig. 1c), and podocin was subsequently recognized as a band with an apparent molecular mass of 42 kDa using Western blot analysis. Additional higher molecular weights were also detected, suggesting the presence of podocin oligomers^20^. To further confirm the interaction between podocin and SNX9, endogenous proteins were immunoprecipitated from ADR-treated cultured podocytes using anti-podocin and anti-SNX9 antibodies. Anti-SNX9 antibody precipitated SNX9 and coprecipitated podocin. Conversely, anti-podocin antibody precipitated podocin and coprecipitated SNX9 (Fig. 1d). However, anti-GFP antibody did not precipitate SNX9 or podocin. Therefore, these results could prove the interaction between SNX9 and podocin via the BAR domain of SNX9 in injured podocytes.

**Figure 2:**
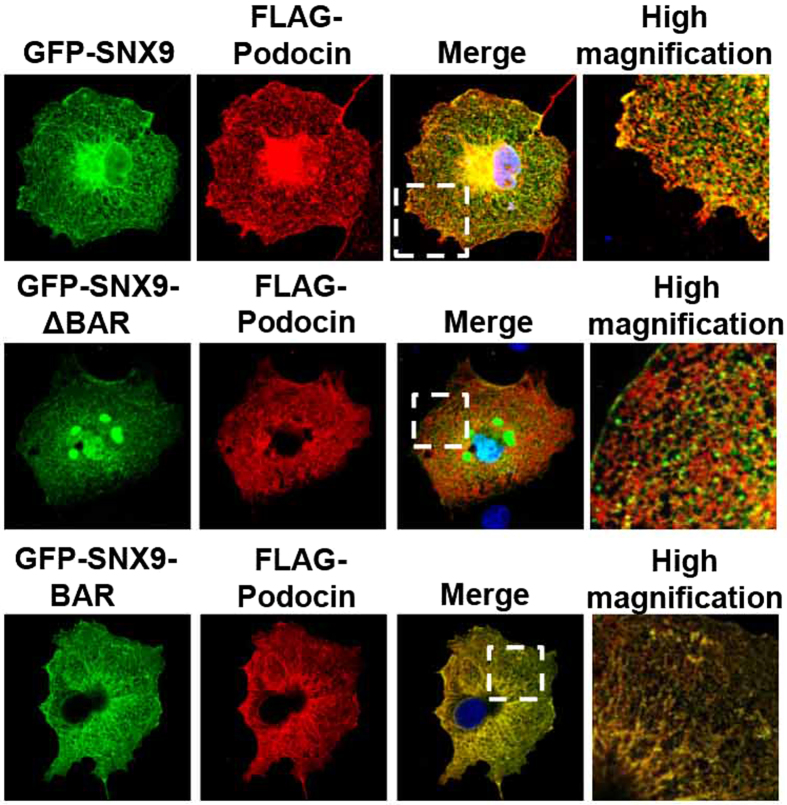
SNX9 co-localizes with podocin in transfected COS7 cells. Immunofluorescence analysis of transiently expressed GFP-tagged SNX9 truncated mutants (green), FLAG-tagged podocin (red) and DAPI (blue) in wild-type COS7 cells. Boxes indicate higher magnification areas presented on the right.

To delineate the functional relevance of the SNX9–podocin interaction, we conducted co-transfection studies of COS7 cells. First, we tested whether SNX9 would interact with podocin in COS7 cells and found that following transient transfection, GFP-SNX9 did indeed co-localize with FLAG-podocin. Next, to confirm the results of our co-immunoprecipitation assay, we co-transfected cells with truncated GFP-SNX9 mutants and FLAG-podocin. Again, GFP-SNX9-BAR co-localized with FLAG-podocin. In contrast, when cells were co-transfected with FLAG-podocin and a GFP-SNX9 mutant lacking the BAR domain, we observed little co-localization (Fig. 2). These data confirm the results of our co-immunoprecipitation assay and verify that SNX9 interacts with podocin via the SNX9 BAR domain.

### Kidneys from ADR-injected mice exhibit high levels of co-localized SNX9 and podocin

**Figure 3:**
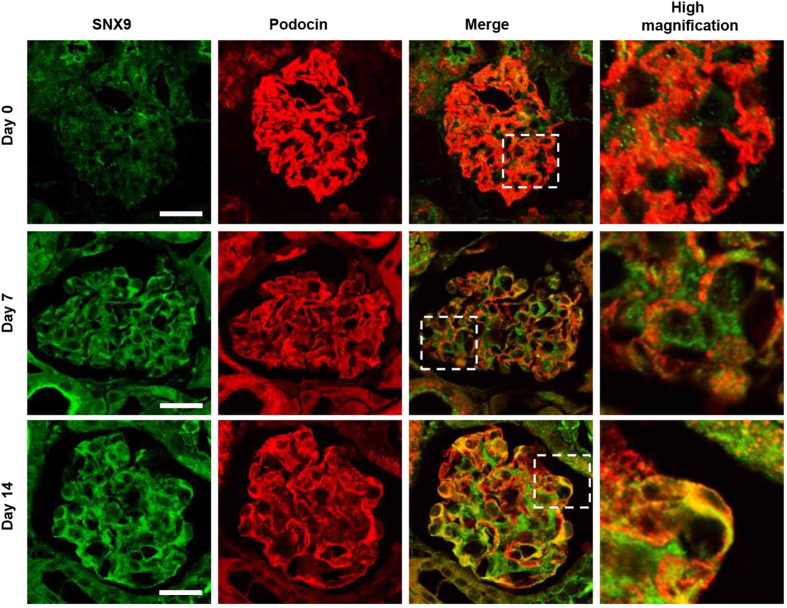
Increased SNX9 expression and co-localization with podocin are detectable in mice with ADR-induced nephrosis. Fluorescent micrographs of glomeruli from mice with ADR-induced nephrosis following immunostaining for SNX9 (green) and podocin (red); merged areas are indicated in yellow. Boxes indicate higher magnification areas presented on the right. Scale bar, 20 μm.

In a previous study, we demonstrated translocation of podocin to the cytoplasm via the endocytosis pathway in injured podocytes^14^. Here, we investigated the potential association of SNX9 with the endocytic translocation of podocin to the cytoplasm in injured podocytes. Using immunofluorescence staining to detect SNX9 expression in kidney samples from normal and ADR-injected mice (Fig. 3), we observed weak SNX9 expression and little co-localization with podocin in the normal mouse kidney (day zero), in contrast to a significant increase in SNX9 expression in the podocyte cytoplasm on days seven and 14 after ADR injection. We further noted that despite a decrease in podocin expression after ADR injection, as described previously^21^, the SNX9 and podocin immunofluorescent signals were merged after ADR injection, especially in the podocyte cytoplasm.

### SNX9 facilitates the translocation of podocin in injured human podocytes

**Figure 4:**
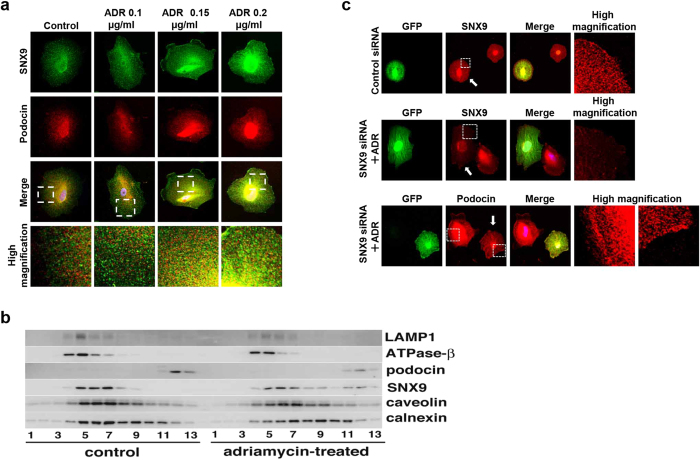
Increased SNX9 expression and co-localization with podocin are detectable in the cytoplasm of ADR-treated WT podocytes, whereas SNX9 KD podocytes exhibit little cytoplasmic expression of podocin. (**a**) Fluorescent micrographs of cultured human podocytes stained with SNX9 (green) and podocin (red) before and after ADR treatment (merged areas are in yellow). DAPI (blue) was used to indicate nuclei. Boxes indicate higher magnification areas presented in the lower panels. (**b**) Western blot analyses of the fractions from control podocytes or podocytes treated with ADR separated on linear OptiPrep gradients (5–25%). Distributions of SNX9 and podocin, as well as marker proteins of plasma membrane (caveolin), endosome/lysosome (LAMP1), mitochondria (β subunit of F1F0-ATPase), and endoplasmic reticulum (calnexin), were examined by western blot analysis. (**c**) Cultured human podocytes were transfected with nonfunctional control siRNA (upper panel) or SNX9 siRNA (middle and lower panels). *Transfected cells,* as decided by *GFP* expression, are indicated by arrowheads. Upper panel: Fluorescent micrographs of control siRNA-transfected podocyte stained with SNX9 (red). Middle panel: Fluorescent micrographs of SNX9 siRNA-transfected podocyte with ADR treatment stained with SNX9 (red). Lower panel: Fluorescent micrographs of SNX9 siRNA-transfected podocyte with ADR treatment stained with podocin (red). DAPI (blue) was used to indicate nuclei. Boxes indicate higher-magnification areas presented on the right.

To evaluate SNX9 expression *in vitro*, we subjected non-treated and ADR-treated cultured human podocytes to immunofluorescence staining (Fig. 4a). Non-treated cultured podocytes showed very little SNX9 expression; in contrast, SNX9 expression was significantly increased in podocytes after ADR treatment. Notably, SNX9 expression increased in a dose-dependent manner. Additionally, we confirmed the co-localization of SNX9 with podocin in podocytes following ADR treatment, which further confirmed the SNX9–podocin interaction.

To study the localization of SNX9 and podocin in ADR-treated podocytes, we performed subcellular fractionation using OptiPrep gradient centrifugation. Podocin remained in fractions 11–13 after ADR treatment. However, SNX9 appeared *de novo* in fractions 9–13 and was distributed in the same fractions where podocin was predominant (Fig. 4b). To investigate the role of SNX9 in injured podocytes, we studied the loss-of-function of SNX9 in ADR-treated podocytes. A decrease in SNX9 expression was found in SNX9 siRNA-transfected podocytes compared with that in non-transfected podocytes (Fig. 4c). SNX9 siRNA-transfected podocytes after ADR treatment exhibited membranous and moderate cytoplasmic expression of podocin, whereas non-transfected podocytes after ADR treatment exhibited strong cytoplasmic and little membranous expression.

### SNX9 co-localizes with endosomal markers in transfected COS7 cells

**Figure 5:**
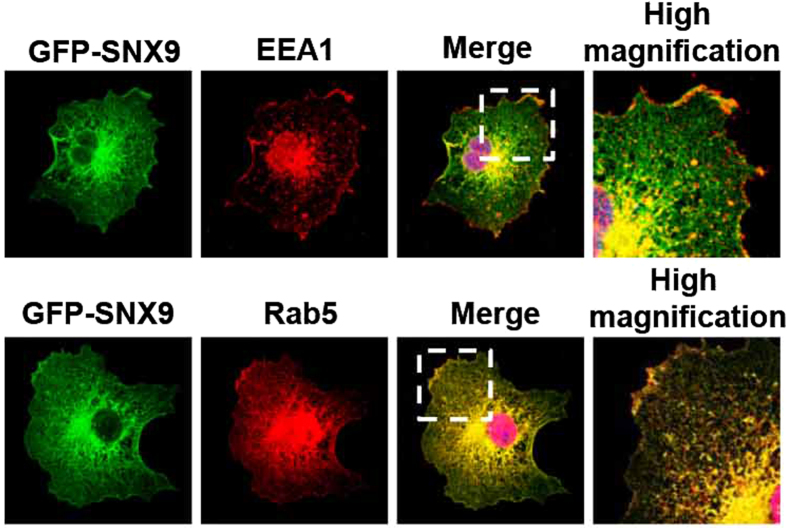
SNX9 localizes to the early endosomes in transfected COS7 cells. Triplicate staining of transiently expressed GFP-tagged SNX9 (green), EEA1 (red, upper panel) or Rab5 (red, lower panel) and DAPI (blue). Boxes indicate higher magnification areas presented on the right.

To confirm the involvement of SNX9 in endocytosis, COS7 cells were transfected with GFP-SNX9 (Fig. 5) and subsequently co-stained with EEA1 and Rab5 (early endosomal compartment marker). Notably, co-localization of SNX9 dots with EEA1 dots was detected in merged figures obtained under high magnification. Furthermore, results show that co-localization of SNX9 with Rab5 was more pronounced.

### SNX9 is detectable in human kidneys affected by IgA nephropathy, membranous nephropathy and focal segmental glomerulosclerosis

**Figure 6:**
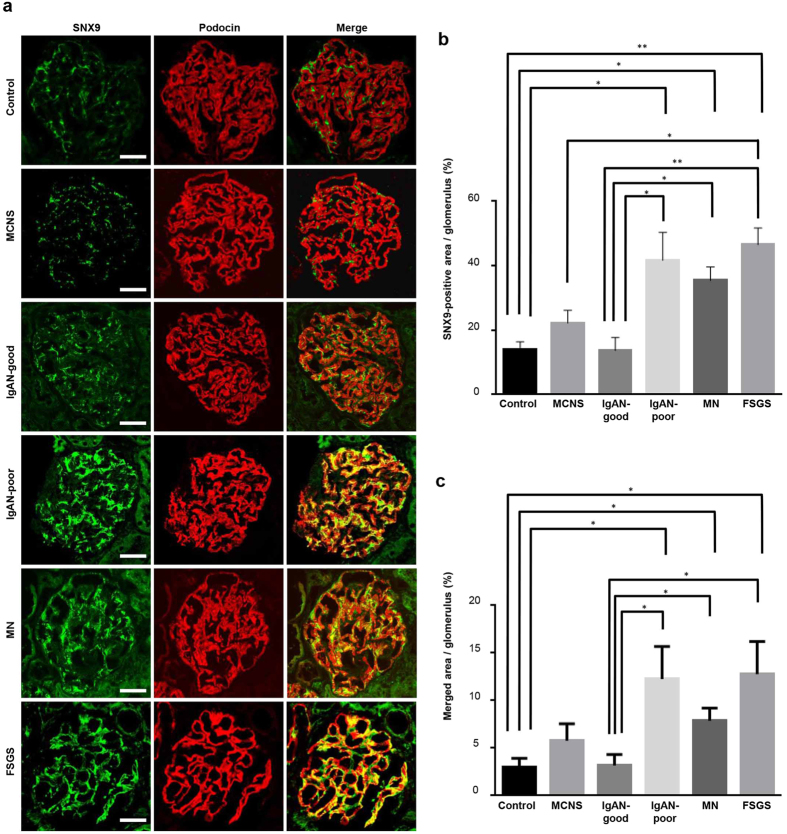
SNX9 is strongly expressed and co-localizes with podocin in the context of severe podocyte injury related to human kidney disease. (**a**) Fluorescent micrographs of glomeruli from human kidney biopsy specimens immunostained with SNX9 (green) and podocin (red); merged areas are in yellow. Kidney samples were diagnosed pathologically. (**b**) SNX9 intensity per glomerulus in human kidney biopsy specimens. More than two glomeruli per patient and six patients per disease were examined, and the SNX9 staining area was automatically quantified using Tissue Studio (Definiens, Munich, Germany; *P < 0.05 or **P < 0.001, one-way ANOVA). (**c**) Intensity of SNX9/podocin merged area per glomerulus in human kidney biopsy specimens. More than two glomeruli per patient and six patients per disease were examined. The SNX9/podocin merged area was automatically quantified using Tissue Studio (Definiens, Munich, Germany; *P < 0.05 or **P < 0.001, Student’s t-test). Scale bar, 50 μm.

To assess SNX9 expression in human kidney glomeruli, we subjected human kidney biopsy specimens to immunofluorescence staining (see Methods). Normal human kidney tissues show moderate SNX9 glomerular staining as shown in The Human Protein Atlas; http://www.proteinatlas.org/. SNX9 expression and co-localization with podocin were significantly higher in specimens from patients with IgA nephropathy with poor prognosis (IgAN-poor), membranous nephropathy (MN) and focal segmental glomerulosclerosis (FSGS); these specimens are associated with severe podocyte injury compared with those from controls, patients with minimal change nephrotic syndrome (MCNS) and patients with IgA nephropathy with a good prognosis (IgAN-good; Fig. 6).

## Discussion

In this study, we identified the protein SNX9 as a novel interaction partner of podocin in podocytes. We detected strong SNX9 expression and co-localization with podocin in ADR-injected mice and samples from human patients with severe glomerular diseases such as IgAN-poor, MN and FSGS, suggesting a pivotal role for SNX9 in podocin endocytosis under pathological conditions.

Sorting nexins have roles in diverse processes such as endocytosis, endosomal sorting and signaling^22^,^23^. Given their fundamental natures, these proteins are associated with diseases in which endosomal function is adversely perturbed, such as Alzheimer’s disease and pathogenic infection^24^,^25^. In this study, SNX9 was found to interact with podocin via its BAR domain; this domain enables the formation of a crescent-shaped homodimer, senses and generates positive membrane curvature and induces membrane tubulation^26^. Furthermore, membrane tubulation via polymeric BAR domain assembly is thought to be regulated by lipid binding^27^. As podocin is a lipid mitochondria protein^17^ that recruits cholesterol to organize the lipid microenvironment of associated ion channel complexes^28^, podocin is thought to have an affinity for the BAR domain, and these structures share a similar polymerizing function. Our data are consistent both with these inferred roles and their functions.

Recent findings have suggested a critical role for endocytosis in podocyte biology, with a particular focus on nephrin^29^,^30^,^31^. However, very little is known about podocin endocytosis, and its precise mechanisms remain unclear. Nevertheless, Shono *et al*. have demonstrated that podocin co-localizes with the coxsackie virus and adenovirus receptor (CAR) at the tight junctions between foot processes in PAN rat kidneys and that podocin facilitates the coalescence of lipid rafts containing CAR and thus promotes dynamic cytoskeletal arrangements^32^. Moreover, podocin and CAR exhibit a diffuse punctate pattern throughout the cytoplasm in COS-7 cells co-transfected with both proteins. In contrast, however, Godel *et al*. demonstrated that a fraction of podocin resides in the CD63/LAMP3-positive late endosomal compartment and has limited co-localized with EEA1 in transiently co-transfected HeLa cells^33^. We recently reported considerable co-localization of both podocin and nephrin with Rab7 and LAMP1 in podocyte-specific cathepsin D knock-out mice (CD^pdKO^), compared with control mice^34^, suggesting that podocin and nephrin are mainly localized in the late endosomes and lysosomes of CD^pdKO^ mouse podocyte cell bodies. Further, electron microscopic examination revealed that cytoplasmic podocin was localized in granular osmiophilic deposits (GRODs).

One previous report has demonstrated that podocin trafficking depends on the raft-mediated, non-classic, clathrin-independent endocytic pathway^33^. Intriguingly, the podocin-related protein flotillin-1 defines a clathrin-independent endocytic pathway^35^, suggesting that podocin not only assembles members of the slit diaphragm, but also orchestrates their internalization via a self-defined pathway^33^. Our group demonstrated the co-localization of podocin with Rab5 in PAN rats, a model of glomerular sclerosis development, indicating that podocin interacts with the CME pathway. We have also previously clarified^15^,^16^ that SNX9 is involved in CME via immunofluorescent analysis of transfected COS7 cells. Specifically, transiently expressed GFP-SNX9 is co-localized with both EEA1 and Rab5, indicating a role for SNX9 in CME. Consistent with our earlier findings^14^, this study demonstrates the involvement of SNX9 in severe podocyte injury and a potential role for this protein as a trigger of podocin endocytosis by CME.

Regarding podocyte endocytosis, Soda *et al*. reported massive proteinuria and kidney failure with histological features suggestive of FSGS in podocyte-specific dynamin-1 and 2 double knock-out mice^36^. This finding supports an important role for dynamin, which is essential to CME, in the maintenance of the renal permeability barrier^37^. Notably, SNX9, which binds directly to dynamin and stimulates dynamin assembly, also stimulates the basal GTPase activity of dynamin and thus potentiates assembly-stimulated GTPase activity on liposomes^38^. The SNX9-dependent recruitment of dynamin to the membrane is regulated by an interaction between SNX9 and aldolase^39^. In other words, SNX9 is required for efficient CME and regulates dynamin activity. In this study, SNX9 expression emerged in the setting of severe glomerular damage, indicating that SNX9 might facilitate podocin endocytosis by regulating dynamin in an attempt at protection from severe damage. Furthermore, ADR-treated SNX9 KD podocytes exhibited membranous and moderate cytoplasmic expression of podocin, whereas non-transfected cell exhibited strong cytoplasmic and little membranous expression. These data indicate that SNX9 is crucial and may facilitate podocin endocytosis in injured podocytes. As ADR induces cell death, it is difficult to obtain sufficient amount of ADR-treated cultured podocytes for western blot analysis. Although few cells survived after ADR treatment, it was possible to perform immunohistochemistry. Therefore, the cells were cultured with a stronger ADR treatment for immunohistochemistry. SNX9 may connect with podocin during the early phase of podocyte injury, as indicated by the subcellular fractionation of cultured human podocytes, and then recruit podocin to cytoplasm during the late phase of podocyte injury, as indicated by immunohistochemistry.

After endocytosis, cargo enters and is sorted in the early endosomes and is either recycled back to the plasma membrane or degraded in the late endosomes and lysosomes^9^. The destinies of slit-diaphragm proteins following endocytosis remains unclear, as cargo can be transferred from early to late endosomes followed by lysosomal degradation, the trans-Golgi network and recycling endosomes^13^. Podocin was found to be internalized with SNX9 in early endosomes, designated by EEA1 and Rab5, and co-localized with the late endosomal proteins Rab7 and LAMP1^34^. SNX9 might promote podocyte injury by degrading podocin in lysosomes, or prevent damage by recycling podocin back to the plasma membrane.

In conclusion, we have demonstrated the emergence of SNX9 expression and consequent internalization of podocin via endocytosis in an ADR-induced nephropathy model, as well as in samples from patients with IgA nephropathy with a poor prognosis, MN and FSGS. SNX9 immunostaining might indicate the degree of podocyte injury. Furthermore, SNX9 may be the key to understanding glomerular injury. Future studies involving SNX9 knockout and overexpression models might clarify the role of SNX9 in podocyte injury.

## Methods

### Yeast two-hybrid screen

The C-terminus of human podocin (amino acids 268–383; GenBank accession number gi: 7657614) was cloned into the bait vector pGBKT7 (BD Biosciences Clontech, San Jose, CA) to create a fusion protein with the GAL4 DNA binding domain; this was subsequently transformed into the yeast strain AH109 as described previously^40^. Briefly, a pre-transformed human kidney MATCHMAKER cDNA library was screened in accordance with the manufacturer’s protocol (MATCHMAKER TwoHybrid System 3; BD Biosciences Clontech). Prey plasmids were isolated, sequenced and retransformed into AH109 cells in combination with the podocin bait construct, a control plasmid (pGBKT7-lamin; BD Biosciences Clontech), or the empty bait vector pGBKT7 to exclude false positives.

### Plasmid constructs

GFP-SNX9 was kindly provided by S. Lance MaCaulay^41^, while GFP-SNX9-BAR, GFP-SNX9-ΔBAR, GFP-SNX9-PXBAR and GFP-SNX9-ΔPXBAR were provided by Sunghoe Chang^18^, and podocin-containing plasmids were provided by K. Schwarz^20^. PCR products were ligated into the FLAG 5a plasmid (Sigma-Aldrich, St. Louis, MO, USA), and all constructs were verified by DNA sequencing.

### Cell culture and transfection

Conditionally immortalized human podocyte cells were a gift from Moin A. Saleem (Bristol Royal Hospital for Children Bristol, Bristol, UK) and were cultured as previously described^42^. Transient transfection of HEK293T and COS7 cells (ATCC) was performed using FuGene 6 Reagent (Roche, Indianapolis, IN, USA) at a 1:3 DNA:Fugene ratio in accordance with the manufacturer’s protocol. GFP-fusion proteins in living cells were analysed using direct fluorescence microscopy.

For ADR-treatment evaluation, cultured podocytes were treated with 0.1–0.2 μg/ml of ADR in a regular medium for 6 h. After treatment, podocytes were washed twice in the medium and then maintained in ADR-free medium for 48 h. As a control, cultured podocytes were treated with normal sterile saline for the same time period and similarly washed. For endogenous co-immunoprecipitation and subcellular fractionation from ADR-treated podocytes, cells were treated with 0.2 μg/ml of ADR in a regular medium for 6 h and then maintained in ADR-free medium for 48 h. Cultured podocytes transfected with control siRNA and SNX9 siRNA were treated with 0.25 μg/ml of ADR in a regular medium for 9 h and then maintained in ADR-free medium for 48 h for immunohistochemistry.

To generate SNX9 KD podocytes, GIPZ lentiviral shRNA system (GE Dharmacon, CO, USA) was used according to the manufacturer’s instructions. In brief, HEK293T cells were transfected with shRNA plasmid DNA containing GFP (GIPZ non-silencing lentiviral shRNA control as a control, V2LHS_114991 as SNX9 KD) to produce lentiviral particles. Differentiated cultured podocytes were transduced with the lentiviral particles.

### Antibodies

Monoclonal mouse Rab5 antibody (#50523; Abcam, Tokyo, Japan), rabbit polyclonal GFP antibody (GFP-Rb-Af2020; Frontier Institute, Ishikari, Hokkaido, Japan), mouse monoclonal FLAG antibody (F1804; Sigma-Aldrich), goat polyclonal EEA1 antibody (sc-6415; Santa Cruz Biotechnology, Dallas, TX, USA), goat polyclonal GST antibody (27–4577; GE Healthcare, Wauwatosa, WI, USA), Alexa Fluor 488-conjugated donkey anti-rabbit IgG antiserum, and Alexa Fluor 555-conjugated goat anti-mouse IgG antiserum (Invitrogen, Carlsbad, CA, USA) were purchased for immunohistochemistry and/or Western blot analysis. Polyclonal rabbit and guinea pig anti-podocin sera have been described previously^20^.

Antibodies against calnexin (C4731; Sigma Aldrich, St. Louis, MO, USA), caveolin (ab2910; Abcam, Cambridge, UK), and LAMP1 (MAB4800; R&D Systems, Minneapolis, MN, USA) were purchased for subcellular fractionation. Antibody against the β subunit of mitochondrial F1F0-ATPase was prepared as described previously^43^.

To generate antibodies against human SNX9, rabbits were immunized with a hemocyanin-conjugated peptide (single letter code, CFGHPQAYQGPATGDD) corresponding to the amino acids of human SNX9, and resulting antibodies were affinity-purified as described previously^44^ (see Supplementary Fig. S1).

### Western blotting and immunoprecipitation

For co-immunoprecipitation of FLAG and GFP fusion proteins, HEK293T cells were grown on a 10-cm dish to approximately 60% confluence, co-transfected and harvested on ice after 48 h with 5 ml of 50 mM EDTA in phosphate-buffered saline (PBS). Cells were pelleted by centrifugation and washed twice with ice-cold PBS. For cell lysis, the pellet was re-suspended in 1 ml of immunoprecipitation buffer (IP; 50 mM Tris [pH 7.5], 150 mM NaCl, 1.0% Triton X100, protease and phosphatase inhibitors) and incubated on ice for 30 minutes. The cell lysate was cleared by centrifugation for five minutes. One millilitre of cell extract was incubated overnight with 50 μl of agarose beads coated with anti-FLAG-M2 antibody (Sigma-Aldrich) at 4 °C. Beads were collected by centrifugation and washed three times with 1 ml of IP buffer for 30 min on a rotator. Bound proteins were eluted by boiling agarose beads in 50 μl of Laemmli buffer at 95 °C for five minutes. Samples were resolved on SDS–polyacrylamide gels, which were then transferred to membranes, and blocked with 5% non-fat milk solution; these were subsequently incubated with the appropriate primary antibodies. Antibodies against FLAG (Sigma) and GFP (Frontier Institute) were used at a 1:1,000 dilution, and HRP-conjugated secondary antibodies (Promega, Madison, WI, USA) were used at a 1:10,000 dilution. Images were scanned using a C-Digit chemiluminescent Western blot scanner, and densitometry analysis was performed using Image Studio Digits software (LI-COR Biosciences, Lincoln, NE, USA). Endogenous co-immunoprecipitations of lysates from ADR-treated podocytes were performed as previously described^40^ using the following polyclonal primary antibodies: anti-podocin, anti–SNX9 and anti-GFP (negative control).

### GST-binding assays

To study the competitive binding of SNX9 to podocin, GST pulldown studies using purified recombinant proteins were performed as described previously^19^. In brief, FLAG-tagged proteins were expressed in HEK293T cells and purified. A total of 1 μg of GST-SNX9 was immobilized on GSH-agarose beads; the beads were washed five times in 1% Triton X100 in PBS, after which 1 μg of purified FLAG-tagged podocin in 500 μl PBS was added. For competition studies, 0, 100, 500, or 1000 ng of purified FLAG-podocin were added. Reactions were incubated under rotation for 2 h at 4 °C, after which the beads were washed five times in PBS. Proteins were eluted in 100 μl of sample buffer and analysed by SDS-PAGE and immunoblotting. Antibodies against GST and FLAG were used at a 1:1,000 dilution. HRP-conjugated secondary antibodies were used at a 1:10,000 dilution.

### Subcellular fractionation

OptiPrep was purchased from Nycomed Pharma (Oslo, Norway). Control human podocytes and ADR-treated podocytes cultured in three 15-cm dishes were detached from dishes using cell scrapers, suspended in PBS and pooled in 50-ml centrifugal tubes. The suspension was centrifuged at 700 × *g* for 5 min. The cell pellet was suspended in 1-ml extraction buffer, containing 5 mM Tes-NaOH (pH 7.4), 0.3 M sucrose and protease inhibitor cocktail (Roche Diagnostics, Indianapolis, IN, USA), and the cells were homogenized by passing the suspension through 26-gauge syringe (10 up–down strokes). Homogenate was centrifuged at 700 × *g* for 5 min. The supernatant (post-nuclear supernatant) was retained. The pellet was re-suspended in 1-ml extraction buffer, homogenized as described above and re-centrifuged at 700 × *g* for 5 min. The pooled post-nuclear supernatant (1.6 ml) was loaded on 10-ml linear OptiPrep gradients (5–25%) that had been prepared according to the manufacturer’s protocol and centrifuged at 150,000 × *g* for 3 h. Fractions of 0.9 ml were collected from the bottom to the top. Aliquots of fractions were treated with SDS-PAGE sample buffer, applied on 10% or 12.5% SDS-PAGE gels and electrophoresed. The separated polypeptides were examined by western blot analyses using antibodies against LAMP1, β subunit of mitochondrial F1F0-ATPase, calnexin, caveolin, podocin and SNX9.

### Immunohistochemistry

Differentiated podocytes and COS7 cells were cultured on collagen type I-coated cover slips. Differentiated podocytes were treated with ADR. Cells were fixed with 4% paraformaldehyde, permeabilised with 0.3% Triton and incubated with blocking solution (2% foetal calf serum [FCS], 2% bovine serum albumin [BSA], 0.2% fish gelatin in PBS) and primary and secondary antibodies. We used 4′,6-diamidino-2-phenylindole (DAPI) as a nuclei marker. For the immunofluorescent staining of ADR mouse kidneys, the kidneys were fixed by perfusion of 4% paraformaldehyde and 20% sucrose in PBS. The fixed kidneys were frozen in optimal cutting temperature compound. Frozen 4-μm-thick sections sections were incubated with primary antibodies specific for SNX9 (anti-SNX9 rabbit polyclonal antibody), podocin (anti-podocin guinea pig polyclonal antibody) and developed with secondary antibodies. The human kidney specimens were fixed with cold acetone for five minutes and frozen 4-μm-thick sections were immunostained in the same manner as described for ADR mice. All images were captured using a confocal laser microscope (FV1000; Olympus, Tokyo, Japan).

### Mouse Model

Female BALB/c mice were purchased from a commercial vendor (Oriental Yeast Co., Ltd., Tokyo, Japan), and ADR nephropathy was induced as previously described^45^. In brief, ADR (doxorubicin hydrochloride; Wako, Osaka, Japan) diluted with 0.9% saline was injected into eight-week-old BALB/c mice via the tail vein at a dose of 11 mg/kg. Age-matched control mice were injected with an equal volume of PBS only. After anaesthesia with sodium pentobarbital (100 mg/kg BW; Dainippon Sumitomo Pharma, Osaka, Japan), mice were euthanized on days seven and 14 after the injection of ADR. All mice were housed under specific pathogen-free conditions in standard animal cages with free access to standard chow and drinking water. All animal handling and experiments were performed strictly in accordance with the recommendations of the guideline for the Care and Use of Laboratory Animals of the Juntendo University Faculty of Medicine. The experimental protocol was approved by the Animal Care and Use Committee of Juntendo University, Tokyo, Japan.

### Renal histology

Mouse kidneys were fixed via perfusion with 4% paraformaldehyde and 20% sucrose in PBS. For the immunofluorescence study, fixed kidneys were frozen in optimal cutting temperature compound. Human kidney specimens were collected from kidney biopsies performed at Juntendo University Hospital, Tokyo, Japan. We analysed samples from two groups of patients with glomerular diseases; those with minor podocyte injuries and those with severe podocyte injuries. MCNS and IgAN-good prognosis cause minor podocyte injury, whereas IgAN-poor prognosis, MN and FSGS cause severe podocyte injury and consequent glomerulosclerosis. As human controls, we used biopsy samples from patients with minor glomerular abnormalities. We diagnosed and classified patients with IgA nephropathy according to the second guideline of IgA nephropathy^46^. All glomeruli in the stained areas of human kidney biopsy specimens were evaluated per patient. For immunostaining, each patient had two to six glomeruli, and six patients in each group were examined. The staining area was automatically quantified using Tissue Studio (Definiens, Munich, Germany)^47^. In brief, the glomerular area was carefully traced by hand and automatically measured. Custom-made image analysis algorithms were applied to the digital slides to automatically detect and quantify the staining areas. Behind a configured action stands a set of algorithms with defined parameters. The respective algorithms were automatically loaded. The staining area/glomerular area ratio was also calculated. This study was conducted according to the Declaration of Helsinki and was approved by the Institutional Review Board of Juntendo University Hospital. Informed consent was obtained from all patients.

### Statistical analysis

All statistical analyses were performed using GraphPad Prism version 6.0 for Windows (GraphPad Software Inc., San Diego, CA, USA). Data are presented as means ± standard errors of the means. Comparisons between groups were analysed using a one-way analysis of variance (ANOVA), or Student’s t-test. Differences with P values < 0.05 were considered significant.

## Additional Information

**How to cite this article:** Sasaki, Y. *et al*. Sorting Nexin 9 facilitates podocin endocytosis in the injured podocyte. *Sci. Rep.* **7**, 43921; doi: 10.1038/srep43921 (2017).

**Publisher's note:** Springer Nature remains neutral with regard to jurisdictional claims in published maps and institutional affiliations.

## Supplementary Material

Supplementary Information
