# Supplementary material for: Sorting Nexin 9 facilitates podocin endocytosis in the injured podocyte
Source: Sci Rep. 2017 Mar 7;7:43921. doi: 10.1038/srep43921 (PMC5339724; doi:10.1038/srep43921)

Sorting Nexin 9 facilitates podocin endocytosis in the injured podocyte

Yu Sasaki[1], Teruo Hidaka[1], Takashi Ueno[2], Miyuki Akiba-Takagi[1], Juan Alejandro Oliva Trejo[1,3], Takuto Seki[1,3], Yoshiko Nagai-Hosoe[1], Eriko Tanaka[1,4], Satoshi Horikoshi[1], Yasuhiko Tomino[1], Yusuke Suzuki[1] and Katsuhiko Asanuma[1,3,*]

1 Division of Nephrology, Department of Internal Medicine, Juntendo University Faculty of Medicine, Tokyo, Japan

2 Laboratory of Proteomics and Biomolecular Science, Research Support Center, Juntendo University Graduate School of Medicine, Tokyo, Japan

3 Medical Innovation Center, TMK project, Graduate School of Medicine, Kyoto University, Kyoto, Japan

4 Department of Pediatrics and Developmental Biology, Graduate School of Medicine, Tokyo Medical and Dental University, Tokyo, Japan

Correspondence: *Katsuhiko Asanuma MD, PhD

Medical Innovation Center, TMK Project, Kyoto University Graduate School of Medicine,

53 Shogoin Kawaharacho, Sakyo-ku, Kyoto 606-8507, Japan

Tel: +81-75-366-7437

E-mail: asanuma@tmk.med.kyoto-u.ac.jp

Conflicts of Interest Statement:

None of the authors has a direct conflict of interest relevant to this study.

Beyond the study contents, Katsuhiko Asanuma has received research funding from Mitsubishi Tanabe Pharmaceutical Corporation.

Supplementary figure legends

Supplementary Figure S1. SNX9 is expressed in human cultured podocytes and glomeruli. To assess the distribution of SNX9, an antibody was raised against SNX9. Cultured human podocytes, mouse glomeruli and mouse kidneys were found to express SNX9 to different degrees. SNX9 was dominantly expressed in differentiated podocytes and glomeruli. Quantifications are presented as protein expression ratio normalized to β-actin. Mouse testis lysate was used as a positive control.

Supplementary Figure S1

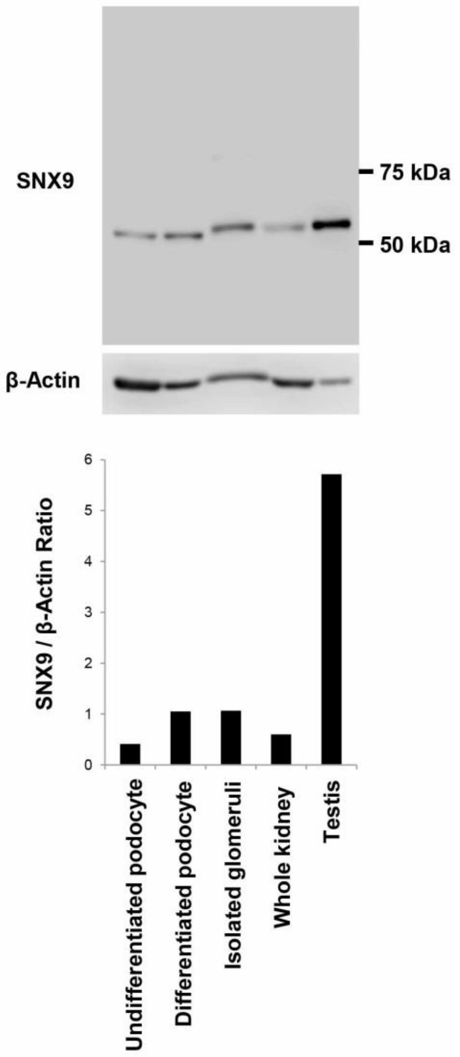

Supplement: Supplementary Information [file srep43921-s1.pdf]
